# WLreg: A new re-parametrization of the Weighted Lindley distribution and its regression model

**DOI:** 10.1371/journal.pone.0324005

**Published:** 2025-06-09

**Authors:** Emrah Altun, Christophe Chesneau, Hana N. Alqifari

**Affiliations:** 1 Department of Mathematics, Bartin University, Bartin, Turkey; 2 Department of Mathematics, University of Caen-Normandie, Caen, France; 3 Department of Statistics and Operations Research, College of Science, Qassim University, Buraydah, Saudi Arabia; Memorial Sloan Kettering Cancer Center, UNITED STATES OF AMERICA

## Abstract

A novel re-parametrization of the weighted Lindley distribution is introduced to develop a regression model suitable for skewed dependent variables defined on ${\mathbb{R}}^{+}$. This new model is called the WL2 regression model. It is shown to outperform existing models such as the gamma, extended gamma, and Maxwell-Boltzmann-exponential regression models. Parameter estimation is performed using the maximum likelihood estimation technique, and the efficiency of these estimates is assessed through a simulation study. An application to a house price data set is presented to highlight the importance of the WL2 regression model. In addition, we propose the WLreg software, accessible via https://bartinuni.shinyapps.io/WLreg, to facilitate the application of the new regression model for practitioners in the field.

## 1 Introduction

### Literature review

The gamma regression model is often used in the analysis of right-skewed response variables, as discussed by [12]. Under the assumption that the response variable conforms to a gamma distribution, [9] developed the gamma regression model, characterized by the simultaneous modeling of the mean and shape parameters. Furthermore, [10] developed a novel diagnostic tool for the analysis of residuals in the gamma regression model. A number of other options have been suggested. These are the weighted exponential (WE) regression by [3], the new extended gamma (NEG) regression by [6], the Maxwell-Boltzmann exponential (MBE) regression by [5], and the Lomax regression by [4].

These types of regression models have important applications in various scientific fields. [18] employed the gamma and quantile regression models to evaluate the factors that affect the medical costs of gastric cancer patients. [13] used the gamma regression model to find the most important factors affecting patient satisfaction. [20] investigated the relationship between environmental factors and the distribution of pelagic fish using gamma error distribution. [23] estimated the conflict-crash relationship using the re-parametrized Lomax distribution proposed by [4]. For further applications of skewed regression models, see [1].

### Contribution

[14] introduced the weighted Lindley (WL) distribution as a generalization of the Lindley distribution proposed by [15]. Bayesian parameter estimation of the WL model was discussed by [2]. More comprehensive work on parameter estimation of the WL distribution has been reviewed by [17]. [7] obtained a new discrete distribution using the WL distribution. The inverse WL distribution was also introduced by [22]. Further interesting work on the WL distribution was done by [16] to make the WL distribution orthogonal to the other shape parameter. [21] introduced the generalized WL distribution using the mixtures of two generalized gamma random variables. [19] introduced the WL regression model using the re-parametrization of [17]. [19] discussed the properties of the WL regression model, including a residual analysis and parameter estimation procedures.

As can be seen from the literature review on the WL distribution, it has been widely investigated by researchers. In this study, we use a different re-parametrization of the WL distribution to propose a new WL regression model. In order not to confuse the two regression models, the model proposed in this study is referred to as the WL2 regression model, and the model proposed by [19] is referred to as the WL1 regression model. The main motivation of the study is to propose a more flexible and efficient model than the existing models for the skewed dependent variables defined on ${\mathbb{R}}^{+}$. The contributions can be summarized as follows:

✓ The WL2 regression model is defined using a novel transformation of the random variable that follows the WL distribution.
✓ The parameter estimation of the WL2 regression model is performed using the maximum likelihood (ML) estimation method, based on ML estimates (MLEs). A simulation study is also carried out to discuss the effectiveness of the MLEs of the model parameters.
✓ To assess the accuracy of the fitted model, a residual analysis is performed, employing the Cox-Snell residuals.
✓ A cloud-based software called WLreg has been developed in the R Shiny environment to allow efficient and widespread use of the WL2 regression model. The WLreg software allows users to easily obtain the results of this model using their own data.

### Organization

This paper is divided into several sections. Sect 2 discusses the re-parametrized WL2 distribution and the associated regression model. It also provides an analysis of the residuals and parameter estimation for the WL2 regression model, complemented by an extensive simulation study. Sect 3 is dedicated to the presentation of empirical results derived from the research. Sect 4 provides detailed information on the use of the WLreg software. The conclusion of the study is summarized in Sect 5.

## 2 WL distribution and regression model

### 2.1 Presentation

We begin with the mathematical background of the WL distribution. First, it is defined by its probability density function (pdf), which is given by

$$f\left(y;\theta ,\alpha \right)=\frac{\theta^{\alpha +1}}{\left(\theta +\alpha \right)\Gamma \left(\alpha \right)}y^{\alpha -1}\left(1+y\right)\exp\left(-\theta y\right),$$ (1)

where $y\in {\mathbb{R}}^{+}$, with θ>0 and α>0 being the scale and shape parameters, respectively, and $\Gamma (\alpha )=\int_{0}^{\infty }t^{\alpha -1}\exp(-t)dt$ is the standard gamma function. The WL distribution can be described as a generalization of the famous Lindley distribution. In particular, it is reduced to the Lindley distribution for α=1. The WL distribution is also a mixture distribution of two independent gamma distributions with the mixing proportion p=θ/(θ+α). The mean and variance associated with the WL distribution are given by

$$\mu =\frac{\alpha \left(\theta +\alpha +1\right)}{\theta \left(\theta +\alpha \right)},$$ (2)

and

$$\sigma^{2}=\frac{\left(\alpha +1\right){\left(\theta +\alpha \right)}^{2}-\theta^{2}}{\theta^{2}{\left(\theta +\alpha \right)}^{2}}.$$ (3)

We are now in a position to introduce the mean-parametrized WL distribution. From Eq 2, we can express α as a function of θ and μ, as follows:

$$\alpha \left(\theta ,\mu \right)=\frac{1}{2}\left(\theta \left(\mu -1\right)+\sqrt{\theta^{2}{\left(\mu +1\right)}^{2}-2\theta \left(\mu -1\right)+1}-1\right).$$ (4)

The notation α(θ,μ) is intended to indicate the dependence of θ and μ in the expression, which will be a crucial point in the proposed regression model. Based on this re-parametrization, the pdf of the WL distribution becomes

$$f\left(y;\theta ,\mu \right)=\frac{\theta^{\alpha \left(\theta ,\mu \right)+1}}{\left(\theta +\alpha \left(\theta ,\mu \right)\right)\Gamma \left(\alpha \left(\theta ,\mu \right)\right)}y^{\alpha \left(\theta ,\mu \right)-1}\left(1+y\right)\exp\left(-\theta y\right).$$ (5)

To indicate the re-parametrization done, the distribution associated with this pdf is called the WL2 distribution. We now consider a random variable *Y* with this pdf, which we refer to using the following stochastic notation: Y~WL2(θ,μ). In particular, its mean is E(Y|θ,μ)= μ.

**Fig 1:**
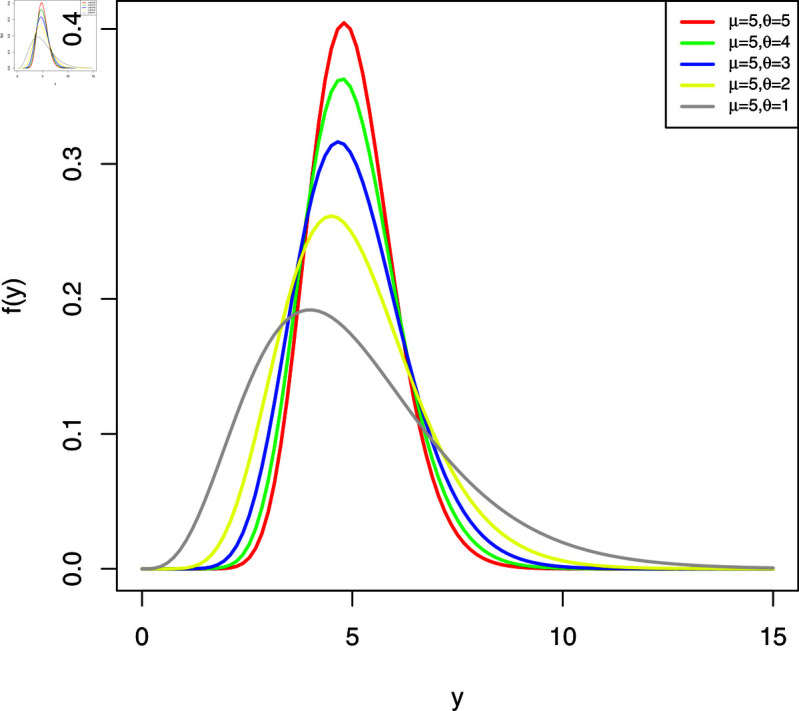
Possible shapes of the pdf (I).

**Fig 2:**
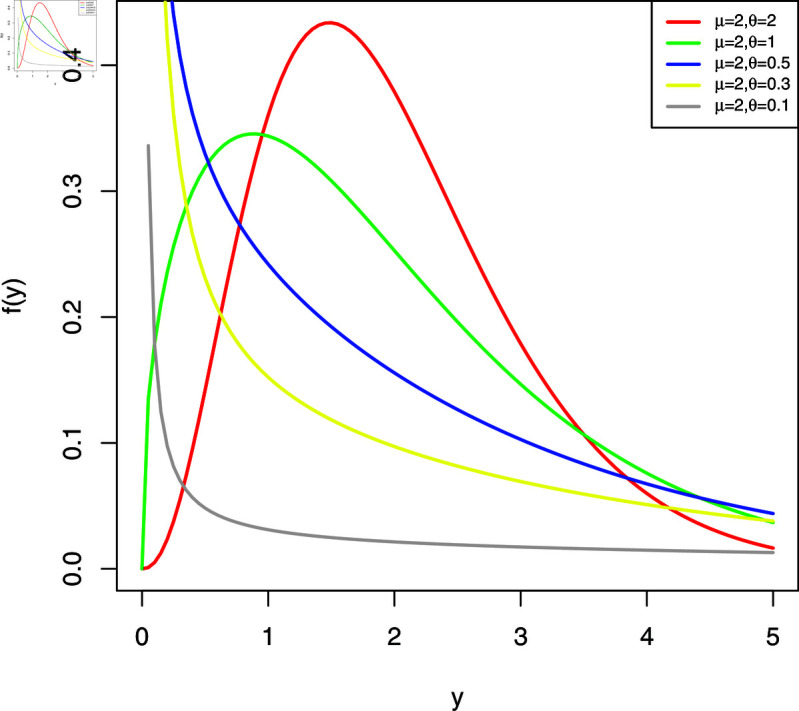
Possible shapes of the pdf (II).

The plots of the WL2 distribution are shown in Figs 1 and 2. The analysis of these plots shows that the distribution is significantly right-skewed, and this skewness becomes more pronounced as the parameter θ is reduced, assuming that the values of μ are held constant.

### 2.2 WL2 regression model

On the mathematical basis of the WL2 distribution, we now discuss the construction of a new WL2 regression model. To do this, we assume that we have a random sample, $y_{1},y_{2},\ldots ,y_{n}$, from $Y_{1},Y_{2},\ldots ,Y_{n}$, where, for any i=1,2,…,n, $Y_{i}\sim WL2\left(\theta ,\mu_{i}\right)$. We recall that $\mu_{i}$ is the mean, assuming that the parameters $\mu_{i}$ and θ are unknown. The introduction of the WL2 model is achieved by using the corresponding link function, which is given by

$$g\left(\mu_{i}\right)=x_{i}^{T}\beta ,$$ (6)

where $\beta ={\left(\beta_{0},\beta_{1},\ldots ,\beta_{k}\right)}^{T}$ is the vector of the regression parameters, *k* is the number of the independent variables, $x_{i}={\left(1,x_{i1},x_{i2},\ldots ,x_{ik}\right)}^{T}$ is the vector representing the values of the covariates, and g:(0,∞)↦ℝ is a link function. The selection of the correct link function is therefore determined by the characteristics of the random variable *Y*.

Using the pdf of the WL2 distribution, given in Eq 5, the log-likelihood function of the WL2 regression model is obtained as

$$\ell \left(\theta ,\beta \right)=\sum_{i=1}^{n}\left(\alpha \left(\theta ,\mu_{i}\right)+1\right)\log\left(\theta \right)-\sum_{i=1}^{n}\log\left(\theta +\alpha \left(\theta ,\mu_{i}\right)\right)+\sum_{i=1}^{n}\log\Gamma \left(\alpha \left(\theta ,\mu_{i}\right)\right)\;+\sum_{i=1}^{n}\left(\alpha \left(\theta ,\mu_{i}\right)-1\right)\log\left(y_{i}\right)+\sum_{i=1}^{n}\log\left(1+y_{i}\right)-n\theta \bar{y},$$ (7)

where $\mu_{i}=\exp\left(x_{i}^{T}\beta \right)$ and $\bar{y}=(1/n)\sum_{i=1}^{n}y_{i}$. The parameter vector, (θ,β), is estimated using the ML approach. The resulting vector is denoted by $\left(\hat{\theta },\hat{\beta }\right)$. For this, we use the Nelder-Mead algorithm defined in the R software. The asymptotic standard errors are obtained using the observed information matrix.

The accuracy of the model is checked by the Cox-Snell (CS) residuals [11]. The CS is defined as

$${\hat{e}}_{i}=-\log\left[1-F\left(y_{i};\hat{\theta },\hat{\beta }\right)\right],$$ (8)

where $F\left(y_{i};\hat{\theta },\hat{\beta }\right)$ is the cumulative distribution function (cdf) of the WL2 taken at *y*_*i*_, with the mention of the dependence in $\hat{\theta }$ and $\hat{\beta }$. If the fitted model accurately represents the data, the CS residuals satisfy $e_{i}\sim \text{Exp}\left(1\right)$.

### 2.3 Simulation

This section looks at the effectiveness of the ML approach in estimating the parameters of the WL2 regression model. The simulation is configured with 1,000 replications. The analysis includes four different sample sizes: 100, 300, 500, and 1,000. The mean vector is defined as $\mu_{i}=\exp\left(\beta_{0}+\beta_{1}x_{i1}+\beta_{2}x_{i2}\right)$, where *x*_1_ and *x*_2_ are drawn from a uniform distribution U(0,1). For the purposes of this analysis, the regression and scale parameters are assigned values of $\beta_{0}=0.5,\beta_{1}=0.5,\beta_{2}=0.5$, and θ=2. The dependent variable *y*_*i*_ is generated based on $\mu_{i}$ and θ through the inverse transform method.

The outcomes of the simulation are summarized in Table 1. They are evaluated on the basis of estimated biases, average estimates (AEs), and mean squared errors (MSEs). It is expected that larger sample sizes will give biases and MSEs that are close to zero, while AEs should be close to the true parameter values. An examination of the results in Table 1 shows that the biases and MSEs are close to zero. In addition, the AEs show stability and remain consistently close to the true parameter values across all sample sizes. These results support the ML approach as an appropriate way to estimate the parameters of the WL2 model.

**Table 1:** Simulation results.

Sample size	Evaluation metrics	$\beta_{0}$	$\beta_{1}$	$\beta_{2}$	θ
100	AE	0.3787	0.6095	0.6209	1.8670
	Bias	-0.1213	0.1395	0.1209	-0.1330
	MSE	1.0043	0.6132	0.6961	0.7966
300	AE	0.3910	0.6003	0.5664	1.8830
	Bias	-0.1090	0.1003	0.0664	-0.1170
	MSE	0.9788	0.4966	0.5301	0.4739
500	AE	0.4363	0.5623	0.5476	1.9283
	Bias	-0.0637	0.0623	0.0476	-0.0717
	MSE	0.8121	0.3927	0.4435	0.3992
1000	AE	0.4994	0.5088	0.5112	1.9822
	Bias	-0.0006	0.0088	0.0112	-0.0178
	MSE	0.7564	0.3276	0.4316	0.3642

## 3 Application

The data set contains 414 observations about the real estate valuation, collected from New Taipei City, Taiwan. The aim is to predict the price of the house using house age (*x*_*i*1_) and number of convenience stores (*x*_*i*2_). The WL2 regression model is compared with the gamma, NEG, MBE and WL1 regression models, as already presented in the first section of the paper.

The gammareg package, developed by [8], is used for the gamma regression model. Once obtained, these values are used as the initial parameter vector for the estimation steps of the WL1, WL2, NEG and MBE regression models. The model in Eq 9 is fitted using the gamma, NEG, MBE, WL1 and WL2 regression models with the mentioned data set:

$$\begin{array}{c} \log\left(\mu_{i}\right)=\beta_{0}+\beta_{1}x_{i1}+\beta_{2}x_{i2},\;i=1,2,\ldots ,n, \end{array}$$ (9)

The MLEs of the parameters and their standard errors are given in Table 2. As all p-values are less than 0.05, the regression parameters are statistically significant. The results of the analysis indicate that an increase in the age of the house is associated with a decrease in the house price, while a greater number of convenience stores is associated with an increase in the house price.

**Table 2:** The estimated coefficients and their respective standard errors.

Parameters		Gamma	NEG	ME	WL1	WL2
$\beta_{0}$	Estimate	3.43785	3.41120	2.77105	3.43786	3.43866
	S.E.	0.03276	0.05976	0.09242	0.03276	0.03229
	p-value	<0.001	<0.001	<0.001	<0.001	<0.001
$\beta_{1}$	Estimate	-0.00706	-0.00706	-0.01377	-0.00706	-0.00763
	S.E.	0.00124	0.00222	0.00332	0.00124	0.00123
	p-value	<0.001	<0.001	<0.001	<0.001	<0.001
$\beta_{2}$	Estimate	0.07309	0.07212	0.04141	0.07309	0.07483
	S.E.	0.00508	0.00926	0.01438	0.00508	0.00451
	p-value	<0.001	<0.001	0.00398	<0.001	<0.001
θ	Estimate	11.18306	4.43660	2.39417	10.24513	0.33465

Table 3 shows the Akaike information criterion (AIC) and Bayesian information criterion (BIC) values of the regression models. The model with the lowest AIC and BIC values is taken as the best model. As can be seen in this table, the model with the lowest AIC and BIC values is the WL2 regression model. Therefore, it was selected as the best model for the data used.

**Table 3:** AIC and BIC values of the models.

Model Selection	Gamma	NEG	MBE	WL1	WL2
−ℓ	1570.439	1685.562	2419.738	1570.322	1549.696
AIC	3148.878	3379.124	4847.476	3148.644	3107.392
BIC	3164.981	3395.227	4863.579	3164.747	3123.495

**Fig 3:**
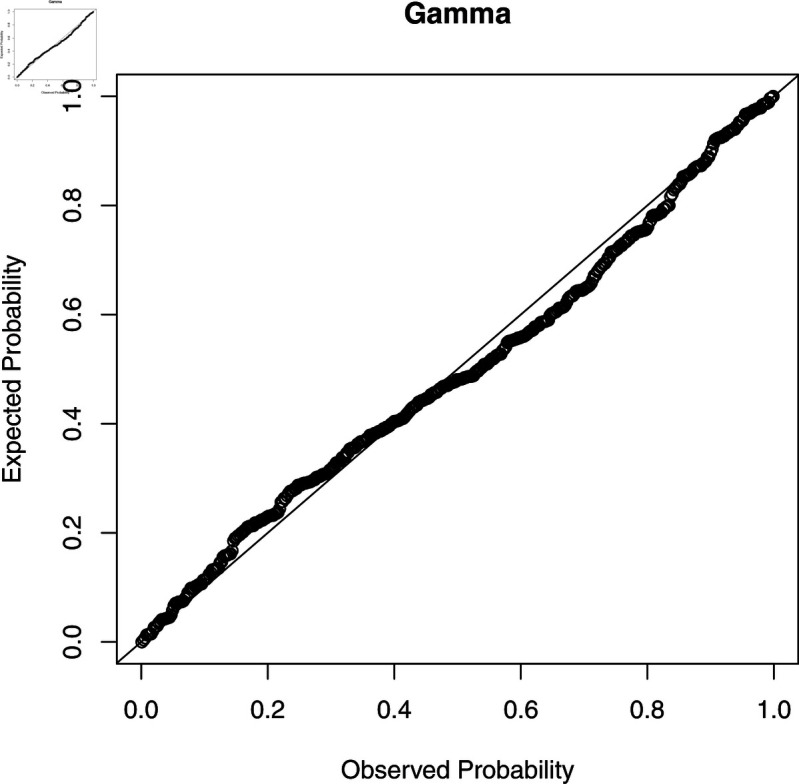
The PP plots of the CS residuals (I).

**Fig 4:**
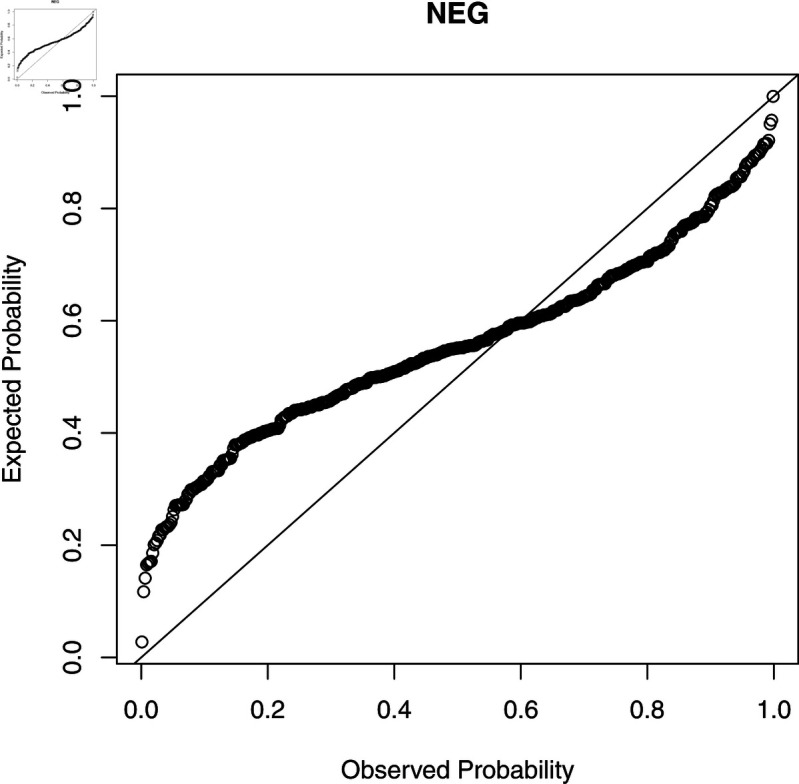
The PP plots of the CS residuals (II).

**Fig 5:**
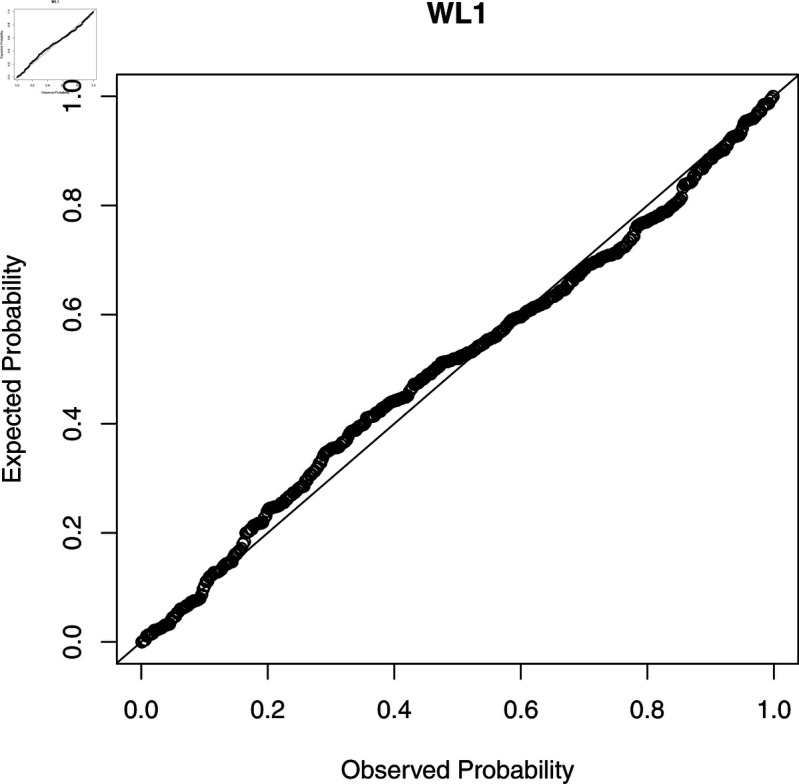
The PP plots of the CS residuals (III).

**Fig 6:**
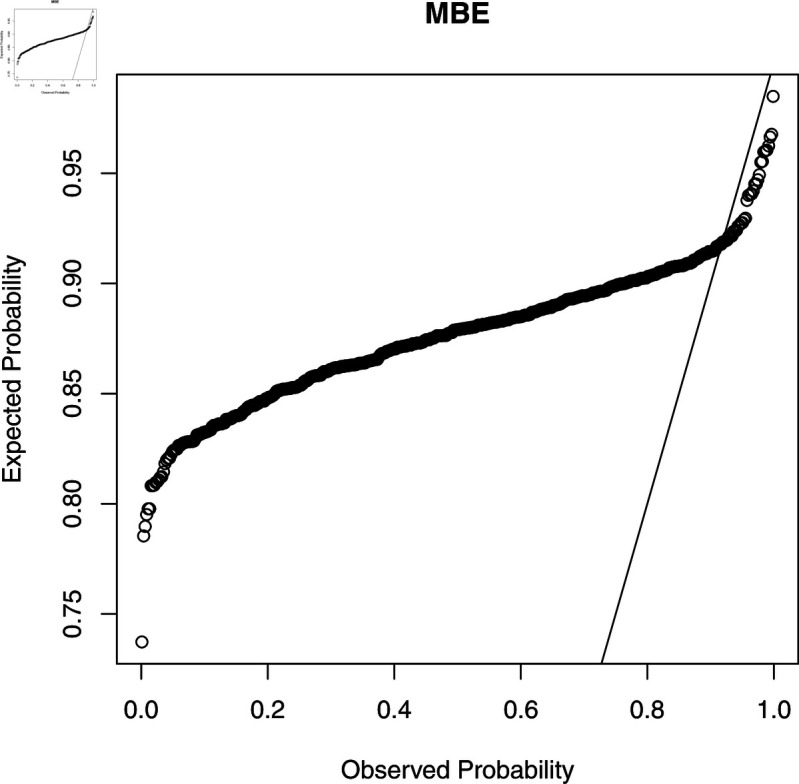
The PP plots of the CS residuals (IV).

**Fig 7:**
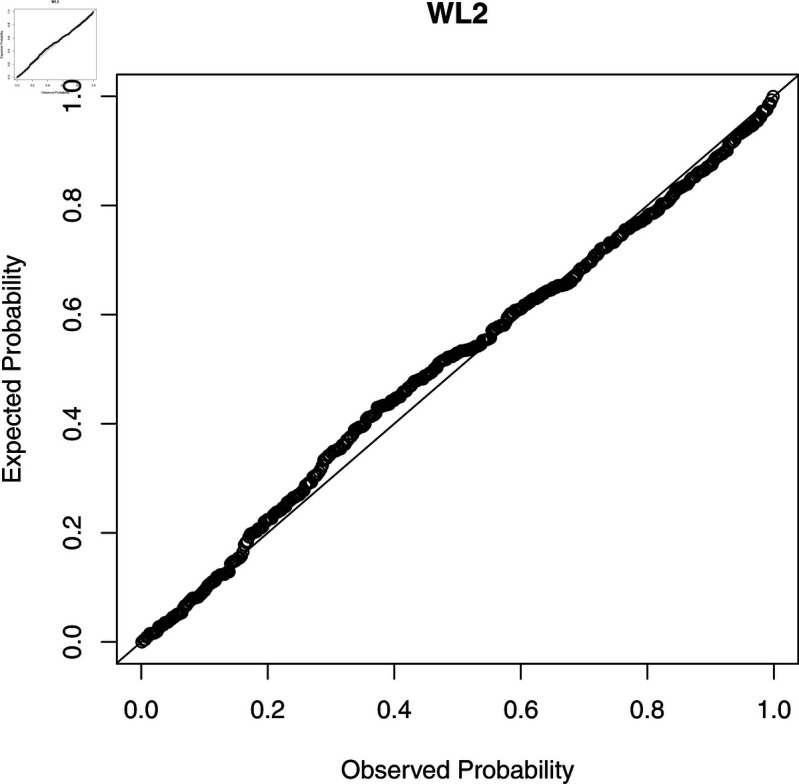
The PP plots of the CS residuals (V).

To check the accuracy of the fitted WL regression model, we compute CS residuals. The probability-probability (PP) plots of the CS residuals are shown in Figs 3, 4, 5, 6, and 7. The Kolmogorov-Smirnov (KS) test is also applied to the CS residuals to check whether or not these residuals follow an exponential distribution. The results of the KS test are given in Table 4. From these results, we can see that the NEG and MBE regression models do not give satisfactory results and the residuals of these models do not follow the exponential distribution. However, the residuals of the gamma, WL1 and WL2 regression models satisfy the assumption for residuals. It is clear that the plotted points of the residuals of the WL2 model are closer to the diagonal line than the other regression models.

**Table 4:** KS test results.

KS	Gamma	NEG	MBE	WL1	WL2
Test statistics	0.057	0.232	0.794	0.057	0.058
p-value	0.141	<0.001	<0.001	0.1402	0.121

The comparison of the models based on the AIC and BIC values may not be sufficient to emphasize the superiority of the WL2 model over the others. Therefore, we use the Vuong non-tested test to compare the WL2 model with other models. [24] proposed a hypothesis to compare the non-tested models. The test statistic is

$$v=\frac{\sqrt{n}\bar{m}}{s_{m}}\sim N\left(0,1\right),$$ (10)

where m¯=∑i=1nmi/∑i=1nminn, $m_{i}=\log\left(f_{2}\left(y_{i},{\hat{\phi }}_{1}\right)\right)-\log\left(f_{1}\left(y_{i},{\hat{\phi }}_{2}\right)\right)$, i=1,…,n, *f*_1_ and *f*_2_ are the pdfs of the two models being compared, and *s*_*m*_ is the standard deviation of $m_{1},\ldots ,m_{n}$. The test statistic in Eq 10 is calculated for all competing models and the results are summarized in Table 5. The null hypothesis is that there is no difference between the models. The alternative hypothesis is that Model I is better than Model II. As reported in Table 5, all p-values are less than 0.05 and the null hypotheses are rejected in favour of the WL2 model in all cases. This evidence supports the conclusion that the WL2 model outperforms the other four competing models.

**Table 5:** Vuong test results.

Model I	Model II	Voung Statistic	p-value
WL2	WL1	15.186	<0.001
WL2	MBE	193.234	<0.001
WL2	NEG	69.991	<0.001
WL2	Gamma	5.036	<0.001

In addition, the F test is used to investigate whether the WL2 model is statistically significant. In the framework of the generalized linear model, the F statistic is calculated by

F=(M1−M2)/(M1−M2)(p2−p1)(p2−p1)M2/M2(n−p2−1)(n−p2−1)~Fp2−p1,n−p2−1, (11)

where *M*_1_ is the deviance of the null model containing only the intercept term and *M*_2_ is the deviance of the fitted model. *p*_1_ and *p*_2_ are the number of the estimated parameters of the two models, respectively. The null hypothesis is that the model is not significant. The F statistic for the WL2 model is obtained as 15.009 and the corresponding p-value is 5.126 × 10^−7^ which is less than 0.05. Therefore, the null hypothesis is rejected and the WL2 model is statistically significant.

## 4 WLreg: Shiny web-tool

**Fig 8:**
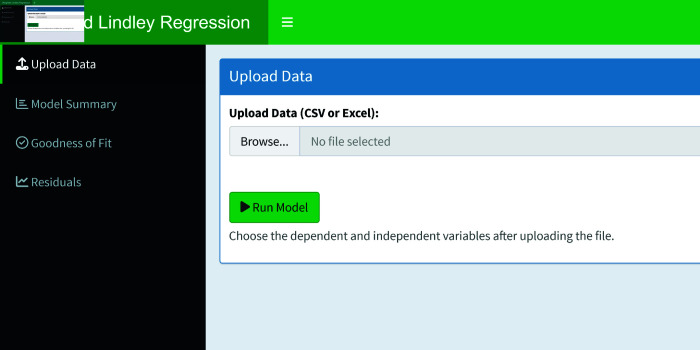
User interface of the WLreg.

In order to make the WL2 regression model easily accessible to researchers, WLreg, a cloud-based software, is being developed. The software is available at https://bartinuni.shinyapps.io/WLreg. WLreg consists of four sections. These are the upload data, model summary, goodness of fit and residuals sections (see Fig 8).

**Fig 9:**
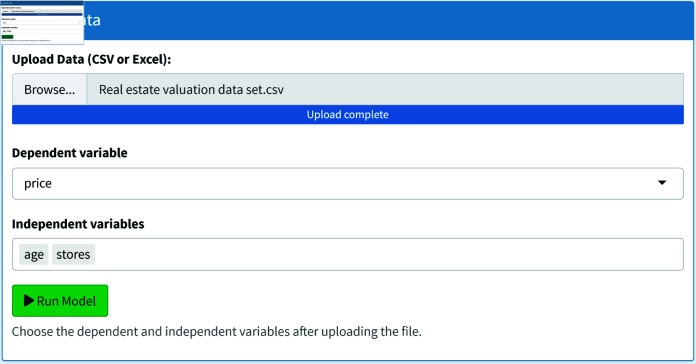
Data upload.

Fig 9 shows the upload data section of the WLreg software. The application results analysed in Sect 3 are given using the WLreg software for the WL2 regression model.

**Fig 10:**
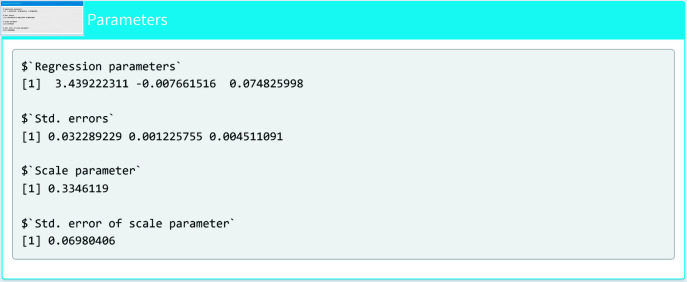
Estimated parameters (I).

**Fig 11:**
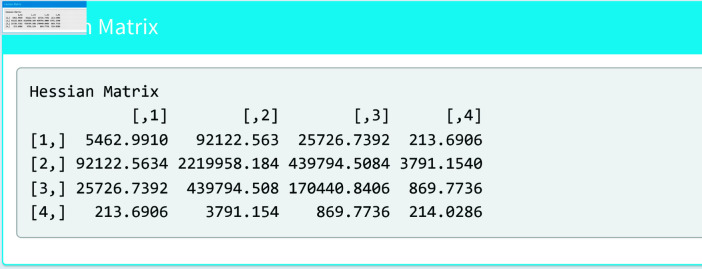
Estimated parameters (II).

Figs 10 and 11 show the summary section of the model. This section shows the estimated parameters, the standard errors and the Hessian matrix.

**Fig 12:**
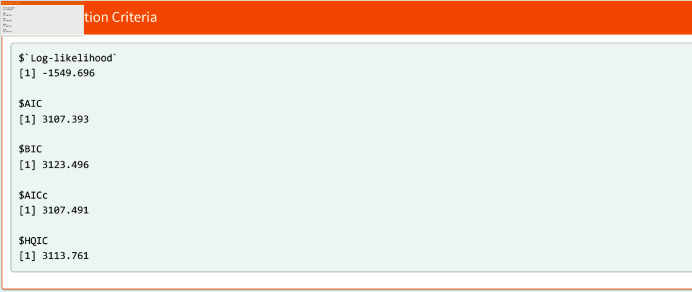
Model selection criteria.

Fig 12 shows the information criteria obtained from the WL2 regression model. These results are important for selecting the best model.

**Fig 13:**
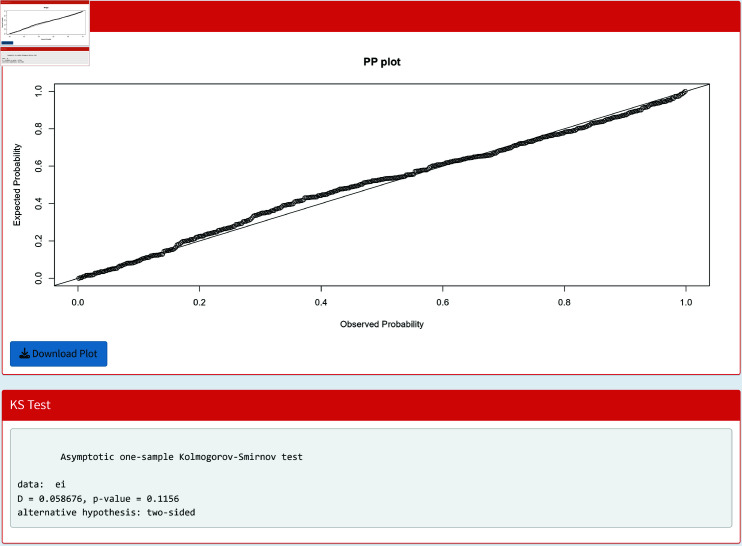
PP plot of the CS residuals with the KS test.

Fig 13 shows the PP plot for CS residuals. The PP plot can be downloaded as a png file by the user. The results of the KS test are also included in this PP plot.

## 5 Concluding remarks and future work

In this paper, the WL2 distribution is obtained by a new parameterisation of the WL distribution. A new regression model has been developed using the WL2 distribution. The efficiency of this regression model is compared with other recently proposed models. The results show that the WL2 regression model gives very successful results. In addition, WLreg software is developed to make the proposed model easy to use.

As a future work, we will introduce a WL2 model with varying dispersion and compare it with its counterparts. The assumption that the dispersion is constant for all observations may not be valid for highly skewed data sets, especially for insurance claims. The mean of the response variable is related to the dispersion parameter via the variance equation. Therefore, as with the mean component, the linear predictor can be used to model the dispersion parameter. The vary-dispersion WL2 model may be more effective in modeling the insurance data sets.
